# An expedient synthesis of 5-*n*-alkylresorcinols and novel 5-*n*-alkylresorcinol haptens

**DOI:** 10.3762/bjoc.5.22

**Published:** 2009-05-19

**Authors:** Kirsti Parikka, Kristiina Wähälä

**Affiliations:** 1 Laboratory of Organic Chemistry, Department of Chemistry, P.O. Box 55, FIN-00014-University of Helsinki, Finland, tel. +358 9 191 50356, fax +358 9 191 50357; 2 (present address:) Department of Applied Chemistry and Microbiology, P.O. Box 27, FIN-00014-University of Helsinki, Finland

**Keywords:** 5-*n*-alkylresorcinols, haptens, microwave assisted synthesis, Wittig reaction

## Abstract

The first synthesis of bioactive long alkyl chain 5-*n*-alkylresorcinols, present in whole grain products, by a novel modification of the Wittig reaction is described. All the main long chain 5-*n*-alkylresorcinols present in rye and wheat, including C_23_ and C_25_ analogues and haptens, which have not been previously prepared, were synthesised. Microwave-promoted reactions of a semi-stabilized ylid and alkanals in water gave good yields in both pressurized and open systems. An alternative microwave-promoted synthesis starting from non-stabilized alkyltriphenylphosphonium salts and 3,5-dimethoxybenzaldehyde worked as well. Aqueous media were suitable for the reactions even if the starting materials were not soluble in water. The 5-*n*-alkylresorcinols are potential biomarkers of whole grain intake, and the new hapten derivatives of 5-*n*-alkylresorcinols will open the way for the immunochemical detection techniques of alkylresorcinols.

## Introduction

**Figure 1:**
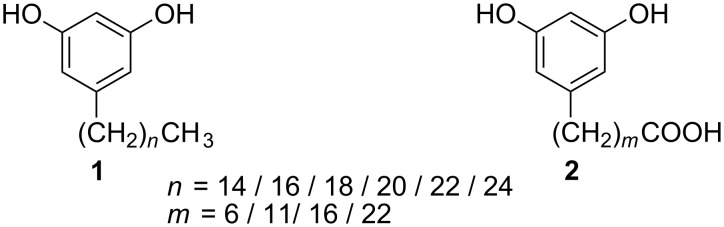
5-*n*-Alkylresorcinols **1** and new hapten derivatives **2**.

5-Alk(en)ylresorcinols and related compounds are phenolic lipids present in several families of plants (e.g. Gramineae, Anacardiaceae, Proteaceae) and in some families of bacteria [1]. We have recently shown that 5-*n*-alkylresorcinols (AR, **1**, see Figure 1) act as antioxidants protecting LDL from oxidative damage in in vitro experiments using synthesized pure analogues with varying chain lengths [2]. In addition, AR have various biological effects including antimutagenic activity [3–6], antibacterial properties [7], inhibition of enzymes [8–12] and interaction with biological membranes by incorporation to the membrane structure [13–14]. Whole grain rye and wheat products, linked to a healthy diet, are the most important dietary source of AR [1]. According to animal and human studies, these compounds are absorbed and at least partially metabolised, and due to their presence in significant amounts in whole grain products they are currently investigated as highly potential biomarkers of whole grain intake [15–21].

However, AR are not generally nor readily available. Thus an efficient preparation method is needed for various analytical, metabolic or bioactivity investigations. The critical step in the synthesis of long alkyl chain (≥C_15_) AR is the formation of C–C bond between the aromatic ring and the alkyl chain. The previously reported multistep syntheses utilize techniques that are time-consuming, require an inert atmosphere and give highly variable overall yields. Undoubtedly, the most common approach has been Grignard or alkyllithium techniques starting from 3,5-dimethoxybenzaldehyde (18–48% yields whenever reported) [22–26]. Additional synthetic methods have been developed for shorter chain AR (<C_15_), such as the aromatization of cyclohexane derivatives in 61–66% yield [27]. A recent Wittig approach utilises the ozonolysis product of a pentadecylresorcinol (C_15_) and odd carbon chain ylids, but its use is limited because of the poor availability of odd carbon chain alkyl bromides [28]. The synthesis of hapten derivatives of alkylresorcinols **2**, potential compounds in the development of immunochemical analysis techniques, has not been reported previously.

Only few papers have reported Wittig reactions in water without an organic solvent, although the related Horner-Wadsworth-Emmons reactions, using ester enolate type stabilized phosphonate ylids, have often been conducted in aqueous solutions [29]. The existing cases have mostly been targeted for the preparation of stilbenes from benzyltriphenylphosphonium salts and aryl aldehydes [30–31] and also include reactions of stabilized ylids with aryl aldehydes or short alkyl chain alkanals [32–36] and the preparation of *o*- and *p*-nitrostyrenes from the highly reactive formaldehyde [37]. To provide water solubility more generally, aryl modified phosphonium salts, carrying a –COOH group [38] or PEG attachments [39], have been developed but require extensive synthetic work. Previously, non-stabilized alkylphosphonium salts have appeared much less amenable than the benzyl analogues. A single paper describes the synthesis of two 1-phenylalkenes in 20–30% yield using benzaldehyde and a CH_2_Cl_2_/H_2_O solvent [31].

We report here a fast and efficient synthesis of the long chain 5-*n*-alkylresorcinols **1** and the new potential hapten derivatives of 5-*n*-alkylresorcinols **2**, ready for the development of antibodies. We also report that the use of microwave (MW) irradiation brings major benefits as regards reaction times and yields in aqueous Wittig reactions. Both semi-stabilized and non-stabilized ylids provide an expedient entry to 5-(1-alkenyl)resorcinols, readily converted to AR and AR haptens.

## Results and Discussion

We approached the Wittig synthesis of the precursors of AR and AR haptens from two aspects choosing semi-stabilized and non-stabilized benzylphosphonium or alkylphosphonium ylids (from salts **3** and **4**,) as starting materials with **5** or **6**, respectively. Water or a mixture of water and an organic solvent were used as a reaction medium with K_2_CO_3_. MW irradiation was used to speed up reaction rates after the preliminary experiments under conventional heating were found to require very long reaction times.

**Scheme 1:**
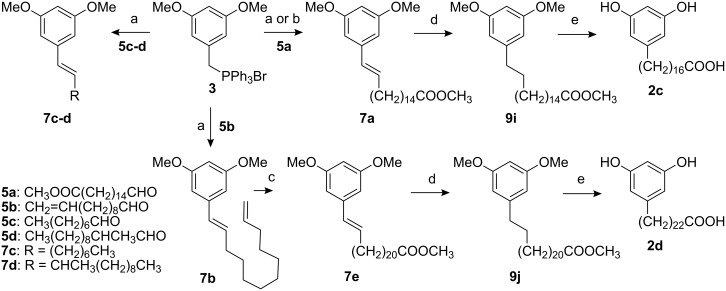
Synthesis of AR derivatives and haptens. a) sealed vessel: MW, 0.1M K_2_CO_3_, 100–150°C, 100–150 W, 4–9 bar, 66–81%; b) open vessel: MW, 0.1M K_2_CO_3_, 80 °C, 50–150W, 89%; c) 9-BBN, K_3_PO_4_, Pd(PPh_3_)_4_, I−(CH_2_)_10_COOMe, 26%; d) H_2_, Pd/C, CH_2_Cl_2_, 90–91%; e) HBr, reflux, 77–79%.

For the reactions of **3** and **5**, 0.1 M K_2_CO_3_ was an optimal solvent. The reactions were performed in 3 or 10 min yielding 66–89% of **7a**–**d** (Scheme 1, Table 1). The methyl group at C-2 of **5d** hindered the reaction and required the use of an increased amount of **3** to give a yield of 66%.

**Table 1:** Wittig reactions performed in 0.1M K_2_CO_3_ or DMSO/H_2_O.

Phosphonium salt	Aldehyde	Product	Time (min)	Yield % (Open vessel)	Yield % (Pressure vessel)	Solvent

**3**	**5a**	**7a**	3	89	81^a^	0.1 M K_2_CO_3_
**3**	**5b**	**7b**	3	–	78	0.1 M K_2_CO_3_
**3**	**5c**	**7c**	3	–	77	0.1 M K_2_CO_3_
**3**	**5d**	**7d**	10	–	66^b^	0.1 M K_2_CO_3_
**4a**	**6**	**8a**	5	81	–	DMSO/H_2_O
**4a**	**6**	**8a**	5	–	48	sat. K_2_CO_3_
**4b**	**6**	**8b**	5	78	–	DMSO/H_2_O
**4c**	**6**	**8c**	5	75	–	DMSO/H_2_O
**4d**	**6**	**8d**	5	76	–	DMSO/H_2_O
**4e**	**6**	**8e**	5	70	–	DMSO/H_2_O
**4f**	**6**	**8f**	5	68	–	DMSO/H_2_O
**4g**	**6**	**8g**	5	68	–	DMSO/H_2_O
**4h**	**6**	**8h**	5	65	–	DMSO/H_2_O

^a^4 bar pressure, otherwise 9 bar. ^b^3 equiv of the phosphonium salt was used in the reaction.

**Scheme 2:**
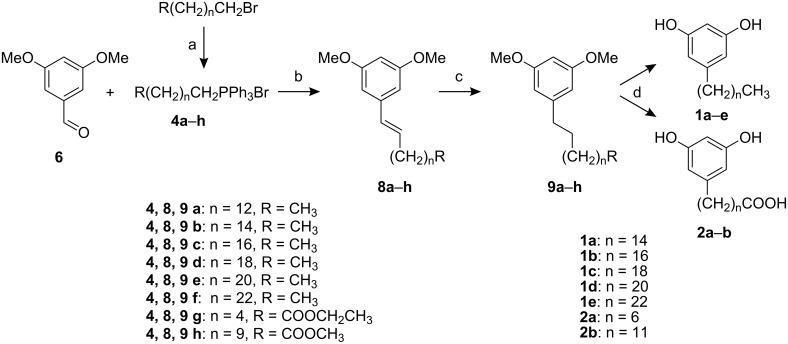
Synthesis of 5-*n*-AR and haptens. a) PPh_3_, toluene, reflux, 79–87%; b) open vessel: MW, DMSO/H_2_O 10:1, K_2_CO_3_, 130–150 °C, 150 W, 65–81%; c) H_2_, Pd/C, CH_2_Cl_2_, 90–97%; d) BBr_3_, 80–84%.

The reactions of **4** and **6** required the presence of an organic solvent (Scheme 2, Table 1), as the yield was not satisfactory in the different K_2_CO_3_ solutions tested (0.1 M/1 M/5 M/saturated). Of those, the saturated solution gave less than 50% at best. A DMSO/H_2_O solution was found to be optimal for these reactions giving the products **8** in 65–81% yield. In both approaches, MW heating shortened the reaction time to minutes and increased the yield (e.g. products **7a** and **8e**) in comparison with the reactions performed under conventional conditions (refluxing several hours in e.g. dioxane/H_2_O/K_2_CO_3_).

The reactions were performed in either open vessels or sealed pressure vessels. The yields of the reactions of the alkylphosphonium salts **4** in sealed vessels were not good due to degradation of the ylids under high pressure (ca. 9 bar). In contrast, reaction of the semi-stabilized benzylic ylid from **3** gave good yield under pressurized conditions, as well as in the open vessel (product **5**, Table 1). A one-pot procedure was investigated using alkyl bromide, PPh_3_ and **6** in various organic solvents but remained unsuccessful.

Mixtures of *cis* and *trans* isomers **7a**–**d** and **8** had *Z*/*E* ratios varying from ca. 40:60 (**8**) to ca. 80:20 (**7a**, both in open and sealed vessel) according to ^1^H NMR and GC-MS. As the ultimate targets were the C=C reduced AR, the lack of stereochemical control was of no consequence. Following the Wittig reaction, catalytic hydrogenation and demethylation gave AR and AR haptens in ca. 40% overall yield. The Wittig product **7b** was a practical starting material for the C_23_ hapten (**2d**), for which commercial alkanal or alkyl bromide precursors are not available (Scheme 1). **5a** was readily synthesised by Swern-type oxidation (see Supporting Information File 1).

The Wittig reagents were poorly soluble in water. Nevertheless, functional groups enhancing their solubility, such as previously reported –COOH or PEG attachments [38–39], were not necessary. Both the yields of the preliminary experiments without MW irradiation and the yields of the MW promoted reactions were superior to those reported previously (20–30%) for Wittig reactions of non-stabilized ylids [31].

The monitoring of AR from biological samples, including human plasma [15], human and animal ileostomy fluids [16–17] and e.g. perirenal adipose tissue [18], has mainly been performed by gas chromatography-mass spectrometry (GC-MS) requiring time-consuming sample preparation such as extraction, derivatisation, chromatography, and special equipment. Compared with this, immunochemical techniques would be relatively straightforward being rapid and suitable for screening purposes in large populations. In the development of immunoanalytical methods, haptens are needed for the preparation of immunogens, which produce specific antisera. Due to the lack of the required haptens, immunoassay has not yet been used in the analysis of AR.

For the haptens prepared, four different alkyl chain lengths were chosen to represent AR with a very short, medium length and long alkyl chain, of which C_17_ and C_23_ are equivalent to the AR present in whole grains and whole grain products. The shorter chain length AR are present, for example, in the plant families Proteaceae (chain lengths C_9_, C_11_) or Anacardiaceae and Primulaceae (C_13_) [1]. The non-polar and hydrophobic character of AR increases as the alkyl chain length grows, which may lead to differences in reactivity.

## Conclusion

We have shown that the MW catalyzed reactions of semi-stabilized ylids and alkanals give good yield in pressurized or open systems and without organic solvent, even if they are not soluble in water, being thus practical starting materials for alkylresorcinols and related compounds. An alternative MW catalyzed synthesis route where 3,5-dimethoxy benzaldehyde and unstabilized alkyltriphenyl phosphonium ylids react in DMSO/H_2_O works as well and alkylresorcinol precursors that do not have a commercially available alkanal starting material can be synthesized rapidly and efficiently by way of this approach. The procedure is suitable for all the long chain 5-*n*-alkylresorcinols, including the C23:0 and C25:0 analogues, for which synthesis has not been reported previously. Functional groups enhancing the solubility in water are not necessary in the MW promoted Wittig reactions of the long alkyl chain reactants. Thus an efficient preparation method of 5-*n*-alkylresorcinols and their hapten derivatives was developed. There was no need for dry solvents or inert atmosphere.

## Supporting Information

File 1 Experimental and data
