# Supplementary material for: An expedient synthesis of 5-n-alkylresorcinols and novel 5-n-alkylresorcinol haptens
Source: Beilstein J Org Chem. 2009 May 19;5:22. doi: 10.3762/bjoc.5.22 (PMC2707013; doi:10.3762/bjoc.5.22)
Supplement: File 1 — Experimental and data [file Beilstein_J_Org_Chem-05-22-s001.doc]

**Experimental and data**

**for:**

An expedient synthesis of 5-*n*-alkylresorcinols and novel 5-*n*-alkylresorcinol haptens

Kirsti Parikka^1,2^ and Kristiina Wähälä^*,1^

Address: ^1^ Laboratory of Organic Chemistry, Department of Chemistry, P.O. Box 55, FIN-00014-University of Helsinki, Finland, tel. +358 9 191 50356, fax +358 9 191 50357 and ^2^(present address:) Department of Applied Chemistry and Microbiology, P.O. Box 27, FIN-00014-University of Helsinki, Finland

Email: Kristiina Wähälä* - [Kristiina.Wahala@helsinki.fi](mailto:Kristiina.Wahala@helsinki.fi)

*Corresponding author.

General

The reagents were purchased from Aldrich except ethyl 7-bromoheptanoate, which was purchased from Pfaltz & Bauer. Methyl 11-iodoundecanoate (Scheme 1, step c) was prepared according to literature [1]. The microwave assisted Wittig reactions were performed using a CEM Discover® system. NMR spectra of the synthesized compounds were obtained with a Varian 200 or 300 MHz spectrometer using tetramethylsilane as an internal standard. Mass spectra were obtained with a JEOL JMS SX102 mass spectrometer operating at 70 eV (EI), or a Varian Saturn 2000 instrument (GC-MS, EI), or a Bruker microTOF spectrometer (ESI). Melting points were determined in open capillary tubes with a Büchi Melting Point B-545 apparatus and are uncorrected.

General procedure for the preparation of the phosphonium salts 3 and 4

PPh_3_ (5.0 mmol) was added to 3,5-dimethoxybenzyl bromide (4.2 mmol) in dry acetone (40 ml) or to an alkyl bromide (4.2 mmol) in dry toluene (5–10 ml). The solution was refluxed overnight. The products **3** and **4a–f** were crystallized by addition of Et_2_O (**3**, 91%, mp 269 °C; **4a–f**, 71–87%, mp **4a** 97 °C, **4b** 101 °C, **4c** 101 °C, **4d** 102 °C, **4e** 106 °C, **4f** 104 °C). Washing with Et_2_O gave **4g** and **4h** as amorphous solids (**4g**, 79%; **4h**, 71%). **3**: ^1^H NMR (300 MHz, CDCl_3_): δ 3.55 (s, 6H, OCH_3_), 5.33 (d, 2H, *J* = 14.4 Hz, CH_2_P), 6.29–6.31 (m, 1H, ArH) 6.35 (t, 2H, *J* = 2.2 Hz, ArH) 7.61–7.81 (m, 15H, ArH); ^13^C NMR (300 MHz, CDCl_3_): δ 30.7, 31.3, 55.5, 101.4, 109.2, 109.3, 117.3, 118.4, 129.2, 130.0, 130.2, 134.5, 134.6, 135.0, 160.7. ^1^H NMR and ^13^C NMR of the products **4a–f** were very similar but consistent with the change in chain length and in agreement with the literature [2]. **4a**: ^1^H NMR (200 MHz, CDCl_3_): δ 0.82–0.86 (m, 3H, CH_3_), 1.18–1.26 (m, 20H, (CH_2_)_10_), 1.57–1.63 (m, 4H, (CH_2_)_2_), 3.81–3.89 (m, 2H, CH_2_P), 7.68–7.86 (m, 15H, ArH); ^13^C NMR (200 MHz, CDCl_3_): δ 14.1, 22.3, 22.7, 23.3, 29.2, 29.3, 29.5, 29.6, 30.3, 30.6, 31.9, 117.4, 119.1, 130.5, 130.7, 133.5, 133.7, 135.1. ^1^H NMR and ^13^C NMR of the products **4g–h** were very similar but consistent with the change in chain length. **4g**: m/z (ESI): 461 (M^+^ − Br). **4h**: ^1^H NMR (300 MHz, CDCl_3_): δ 1.17–1.25 (m, 10H, (CH_2_)_5_), 1.59–1.65 (m, 6H, (CH_2_)_3_), 2.28 (t, 2H, *J* = 7.5 Hz, CH_2_COOCH_3_), 3.66 (s, 3H, OCH_3_), 3.77–3.85 (m, 2H, CH_2_P), 7.69–7.89 (m, 15H, ArH); ^13^C NMR (300 MHz, CDCl_3_): δ 22.5, 22.8, 23.1, 24.9, 28.1, 29.1, 29.3, 30.3, 30.5, 34.1, 51.5, 118.0, 119.1, 130.4, 10.6, 132.1, 133.3, 133.4, 133.7, 133.8, 135.0, 174.7; m/z (ESI): 419 (M^+^ − Br).

Methyl 15-formylpentadecanoate (5a)

16-Hydroxyhexadecanoic acid (3.7 mmol, 1.0 g) was stirred in a 100 ml flask with 50 ml of methanol and 1 ml of H_2_SO_4_ overnight. The solution was concentrated to a volume of 5–10 ml and diluted with water. The crystalline product was filtered to give pure methyl 16-hydroxyhexadecanoate (95%). ^1^H NMR (300 MHz, CDCl_3_): δ 1.22–1.30 (m, 20H, (CH_2_)_10_), 1.54–1.60 (m, 6H, (CH_2_)_3_), 2.30 (t, 2H, *J* = 7.8 Hz, CH_2_COOCH_3_), 3.64 (t, 2H, *J* = 6.6 Hz, CH_2_OH), 3.67 (s, 3H, OCH_3_). Methyl 16-hydroxyhexadecanoate (0.5 g, 1.7 mmol) was stirred with 6 ml of DMSO in a 25 ml two-necked flask under argon gas at 0 °C. P_2_O_5_ was added (0.74g, 5.2 mmol) and the mixture was allowed to come to room temperature. After 1.5 hours the flask was cooled again to 0 °C and 0.8 ml of Et_3_N was added. Stirring was continued for 1 hour at room temperature. 10% HCl was added and the product extracted with CH_2_Cl_2_, washed with brine and purified by flash chromatography (silica gel, CH_2_Cl_2_) giving **5a** (64%). ^1^H NMR (300 MHz, CDCl_3_): δ 1.21–1.29 (m, 20H, (CH_2_)_10_), 1.58–1.64 (m, 4H, (CH_2_)_2_), 2.30 (t, 2H, *J* = 7.6 Hz, CH_2_COOCH_3_), 2.42 (td, 2H, *J* = 7.3 Hz, *J* = 1.8 Hz, CH_2_CHO), 3.67 (s, 3H, OCH_3_), 9.76 (t, 1H, *J* = 1.8 Hz, CHO); ^13^C NMR (300 MHz, CDCl_3_): δ 22.1, 25.0, 29.2, 29.3, 29.36, 29.44, 29.6, 34.1, 43.9, 51.4, 174.3, 202.9; m/z (EI): 81 (7), 95 (10), 135 (7), 191 (12), 209 (10), 235 (12), 253 (25), 284 (M^+^, 100%).

General procedure for the preparation of 7a–d

The phosphonium salt **3** (0.25 g, 0.5 mmol), the aldehyde **5** (0.5 mmol) and 3 ml of 0.1M K_2_CO_3_ (aq) were sealed in a pressure tube. The mixture was stirred under microwave irradiation and external cooling. The temperature reached 100 °C in 2 min where it was kept for 3 min (preparation of **7a**) or 150 °C in 2 min where it was kept for 3 min (preparation of **7b–d**). After cooling down, the product was extracted with Et_2_O. Flash chromatography (silica gel, CH_2_Cl_2_) gave the products as mixtures of *cis*/*trans* isomers (**7a**, ca. 80:20 *cis*/*trans* ratio and **7b–d** ca. 60:40 ratio; **7a**, 81%; **7b**, 78%; **7c**, 77%; **7d**, 66%). Alternatively, the phosphonium salt **3** (0.15 g, 0.3 mmol), the aldehyde **5a** (0.071 g, 0.25 mmol) and 0.75 ml of 0.1M K_2_CO_3_ (aq) were stirred under microwave irradiation and external cooling in a 5 ml flask. The temperature reached 80 °C in 0.5 min where it was kept for 3.5 min. After cooling down, the product was extracted with Et_2_O. Flash chromatography (silica gel, CH_2_Cl_2_) gave **7a** (mixture of *cis*/*trans*, ca. 80:20 ratio, 89%). The mass spectra of the *cis*/*trans* mixtures were very similar with each other but consistent with the change in the chain length. **7a**: m/z (EI) 151 (30%), 152 (100), 177 (30), 191 (40), 417 (50), 418 (95, M^+^), 419 (15). 7: m/z (EI) 151 (20%), 152 (100), 165 (10), 177 (30), 191 (70), 261 (10), 262 (60, M^+^), 263 (7). 11a: m/z (EI) 151 (30%), 152 (100), 165 (10), 177 (20), 191 (30), 301 (5), 302 (15, M^+^), 303 (5).

Methyl 23-(3,5-dimethoxyphenyl)-22-tricosenoate (7e)

1-(3,5-Dimethoxyphenyl)-dodeca-1,11-diene (**7b**) (mixture of *cis*/*trans*, 0.52 g, 1.7 mmol) was dissolved in dry THF under argon and 3.4 ml of a 0.5 M solution of 9-BBN in THF was added at 0 °C. The solution was stirred at room temperature for 2 h and then methyl 11-iodoundecanoate (0.85 g, 2.6 mmol), Pd(PPh_3_)_4_ (0.058 g, 0.05 mmol) and K_3_PO_4_ (1.08 g, 5.1 mmol) were added. The mixture was refluxed for 4 h. Water was added and the product extracted with CH_2_Cl_2_. Flash chromatography (silica gel, CH_2_Cl_2_) gave **7e** (mixture of *cis*/*trans*, ca. 60:40 ratio, 26%). m/z (EI) 151 (5%), 152 (12), 177 (5), 191 (7), 502 (70, M^+^), 503 (25).

General procedure for the preparation of 8

The phosphonium salt **4** (0.6 mmol), 3,5-dimethoxybenzaldehyde (**6**) (0.09 g, 0.54 mmol) and K_2_CO_3_ (0.14 g, 1.0 mmol) in 1 ml of DMSO and 0.1 ml of H_2_O were stirred under microwave irradiation and external cooling in a 5 ml flask. The temperature reached 150 °C in 2 minutes where it was kept for 5 min (products **8a–f**) or 130 °C in 2 min where it was kept for 5 min (products **8g–h**). After cooling down, the product was extracted with Et_2_O. Flash chromatography (silica gel, CH_2_Cl_2_) gave **8** as a mixture of *cis*/*trans* isomers (**8a–f** ca. 40:60 *cis*/*trans* ratio, **8g–h** ca. 50:50 ratio, **8a–f**, 68–81%; **8g**, 68%; **8h**, 65%). The mass spectra of the *cis*/*trans* mixtures **8a–h** were similar with each other but consistent with the change in chain length. **8a**: m/z (EI) 151 (15%), 152 (50), 177 (30), 191 (35), 346 (M^+^, 90), 347 (30).

General procedure for the preparation of 9

The product 7a, 7c–e or 8a–h (1.0 mmol) was dissolved in dichloromethane (40 ml). Palladium on charcoal was added (10 % w/w; 10% w/w of the starting material) and H_2_ gas introduced to the reaction mixture at atmospheric pressure and room temperature. Filtration of the reaction mixture through Celite® gave pure **9a–j** (90–97%). ^1^H NMR, ^13^C NMR and MS of the products **9a–e** were very similar but consistent with the change in chain length and in agreement with those published elsewhere [3-4]. **9i**: ^1^H NMR (300 MHz, CDCl_3_): δ 1.20–1.30 (m, 22H, (CH_2_)_11_), 1.58–1.66 (m, 4H, (CH_2_)_2_), 2.30 (t, 2H, *J* = 7.6 Hz, CH_2_COOCH_3_), 2.54 (t, 2H, *J* = 7.8 Hz, CH_2_Ar), 3.66 (s, 3H, OCH_3_), 3.78 (s, 6H, OCH_3_) 6.29 (t, 1H, *J* = 2.2 Hz, Ar), 6.34 (d, 2H, *J* = 2.3 Hz, ArH); ^13^C NMR (300 MHz, CDCl_3_): δ 25.0, 29.2, 29.3, 29.4, 29.5, 29.7, 31.3, 34.1, 36.3, 51.4, 55.2, 97.6, 106.5, 145.4, 160.7, 174.4; m/z (EI): 121 (5), 137 (3), 152 (100), 165 (15), 194 (5), 389 (5), 420 (M^+^, 45), 421 (20), 422 (10%). **9j**: ^1^H NMR (200 MHz, CDCl_3_): δ 1.19–1.31 (m, 36H, (CH_2_)_18_), 1.59–1.67 (m, 4H, (CH_2_)_2_), 2.30 (t, 2H, *J* = 7.6 Hz, CH_2_COOCH_3_), 2.54 (t, 2H, *J* = 7.8 Hz, CH_2_Ar), 3.67 (s, 3H, OCH_3_), 3.78 (s, 6H, OCH_3_), 6.29 (t, 1H, *J* = 2.2 Hz, ArH), 6.35 (d, 2H, *J* = 2.2 Hz, ArH); ^13^C NMR (200 MHz, CDCl_3_): δ 25.0, 29.2, 29.3, 29.4, 29.47, 29.54, 29.6, 29.7, 31.3, 34.1, 36.3, 51.4, 55.2, 97.5, 106.5, 145.4, 160.7, 174.4; m/z (EI) 121 (3), 137 (5), 152 (100), 165 (15), 194 (5), 473 (10), 504 (M^+^, 90), 505 (40), 506 (10%). **9g**: ^1^H NMR (200 MHz, CDCl_3_): δ 1.25 (t, 3H, *J* = 7.2 Hz, CH_3_), 1.28–1.36 (m, 6H, (CH_2_)_3_), 1.59–1.64 (m, 4H, (CH_2_)_2_), 2.28 (t, 2H, *J* = 7.5 Hz, CH_2_COOCH_2_CH_3_), 2.54 (t, 2H, *J* = 7.7 Hz, CH_2_Ar), 3.78 (s, 6H, OCH_3_), 4.12 (quintet, 2H, *J* = 7.2 Hz, CH_2_CH_3_), 6.30 (t, 1H, *J* = 2.2 Hz, ArH), 6.40 (d, 2H, *J* = 2.2 Hz, ArH); ^13^C NMR (200 MHz, CDCl_3_): δ 14.3, 25.0, 29.07, 29.13, 31.2, 34.4, 36.3, 55.2, 60.2, 97.6, 106.5, 145.3, 160.7, 174.4; m/z (EI): 121(3), 137 (5), 152 (100), 165 (15), 194 (5), 263 (10), 308 (M^+^, 70), 309 (35), 310 (10%). **9h**: ^1^H NMR (300 MHz, CDCl_3_): δ 1.22–1.30 (m, 12H, (CH_2_)_6_), 1.57–1.63 (m, 4H, (CH_2_)_2_), 2.30 (t, 2H, *J* = 7.5 Hz, CH_2_COOCH_3_), 2.54 (t, 2H, *J* = 7.6Hz, CH_2_Ar), 3.66 (s, 3H, OCH_3_), 3.77 (s, 6H, OCH_3_), 6.29 (t, 1H, *J* = 2.2 Hz, ArH), 6.34 (d, 2H, *J* = 2.1 Hz, ArH); ^13^C NMR (300 MHz, CDCl_3_): δ 25.0, 29.2, 29.3, 29.4, 29.5, 29.6, 31.3, 34.1, 36.3, 51.4, 55.2, 97.6, 106.5, 145.4, 160.7, 174.4; m/z (EI): 121(3), 137 (3), 152 (100), 165 (15), 194 (5), 319 (10), 350 (M^+^, 70), 351 (35), 352 (10%).

General procedure for the preparation of 1 and 2

The dimethyl **9** (0.1 mmol) was refluxed in concentrated HBr (7 ml) for 2–4 hours. Water was added to the mixture, which was extracted with EtOAc. Combined organic layers were dried with Na_2_SO_4_ and evaporated. Alternatively, dimethyl ether **9a–e** (0.7 mmol) was dissolved in dichloromethane (25 ml) and treated with 2.8 ml of 1M solution of BBr_3_ in CH_2_Cl_2_ at –70°C. After 5 hours the solution was allowed to come to room temperature and after addition of water the product was extracted with EtOAc. Purification by flash chromatography (silica gel, CH_2_Cl_2_:EtOAc:MeOH, 70:20:1) and recrystallization from cyclohexane gave **1a–e** as white powders (80–84%, mp **1a** 91 °C, **1b** 92 °C, **1c** 97 °C, **1d** 99 °C, **1e** 100 °C). According to GC analysis, the silylated samples of **1a–e** were ca. 99% pure (silylation: 0.5 ml TMCS:HMDS:pyridine, 1:3:9, was added to 1 mg of **1a–e** and incubated for 1 h at 60 °C). Purification by flash chromatography (CH_2_Cl_2_:MeOH, 3:1) and wash with CHCl_3_ gave **2a–d** as white powders (77–82%, mp **2a** 81 °C, **2b** 97 °C, **2c** 107 °C, **2d** 108 °C). The purity of **2a–d** was checked by TLC which showed one spot (CH_2_Cl_2_:MeOH, 3:1; staining with H_2_SO_4_:EtOH:vanillin, 1 ml:100 ml:3.5 mg).

NMR and MS of the products **1a–e** were very similar but consistent with the change in chain length, and consistent with those previously reported [3,5-6]. **1a**: m/z calcd for C_21_H_36_O_2_ (M^+^): 320.2715; found 320.2704. **1b**: m/z calcd for C_23_H_40_O_2_ (M^+^): 348.3028; found 348.3019. **1c**: m/z calcd for C_25_H_44_O_2_ (M^+^): 376.3341; found 376.3334. **1d**: m/z calcd for C_27_H_48_O_2_ (M^+^): 404.3654; found 404.3645. **1e**: ^1^H NMR (200 MHz, D_6_-acetone): δ 0.85–0.91 (m, 3H, CH_3_), 1.23–1.35 (m, 40H, (CH_2_)_20_), 1.53–1.61 (m, 2H, CH_2_), 2.45 (t, 2H, *J* = 6.8 Hz, CH_2_Ar) 6.18 (s, 3H, ArH), 8.01 (br s, 2H, OH); ^13^C NMR (200 MHz, D_6_-acetone): δ 14.4, 23.3, 28.7–31.0 (overlapping with acetone), 32.1, 32.6, 36.6, 100.9, 107.7, 145.9, 159.3; m/z (EI): 123 (45%), 124 (100), 137 (18), 166 (10), 431 (7), 432 (M^+^, 90), 433 (50); m/z calcd for C_29_H_52_O_2_ (M^+^): 432.3967; found 432.3978. **2a**: ^1^H NMR (300 MHz, DMSO-d_6_): δ 1.23–1.31 (m, 6H, (CH_2_)_3_), 1.46–1.52 (m, 4H, (CH_2_)_2_), 2.19 (t, 2H, *J* = 7.3 Hz, CH_2_COOH), 2.36, (t, 2H, *J* = 7.6 Hz, CH_2_Ar), 6.01 (s, 3H, ArH), 8.94 (br s, 2H, ArOH), 11.85 (br s, 1H, COOH); ^13^C NMR (300 MHz, DMSO-d_6_): δ 24.5, 28.5, 30.6, 33.6, 35.3, 99.9, 106.2, 144.1, 158.1, 174.4; m/z (ESI): 251.1289 (M^+^ − H; C_14_H_19_O_4_ requires 251.1283). **2b**: ^1^H NMR (300 MHz, DMSO-d_6_): δ 1.20–1.28 (m, 14H, (CH_2_)_7_), 1.45–1.51 (m, 4H, (CH_2_)_2_), 2.14 (t, 2H, *J* = 6.7 Hz, CH_2_COOH), 2.35 (t, 2H, *J* = 7.2 Hz, CH_2_Ar), 6.01 (s, 3H, ArH), 8.97 (br s, 2H, ArOH), 11.89 (br s, 1H, COOH); ^13^C NMR (300 MHz, DMSO-d_6_): δ 24.7, 28.6, 28.7, 28.86, 28.93, 30.6, 34.2, 35.2, 99.9, 106.2, 144.1, 158.1, 175.3; m/z (ESI): 307.1915 (M^+^ − H; C_18_H_27_O_4_ requires 307.1909). **2c**: ^1^H NMR (300 MHz, DMSO-d_6_): δ 1.19–1.27 (m, 24H, (CH_2_)_12_), 1.45–1.51 (m, 4H, (CH_2_)_2_), 2.17 (t, 2H, *J* = 7.3 Hz, CH_2_COOH), 2.35 (t, 2H, *J* = 7.6 Hz, CH_2_Ar) 6.01 (s, 3H, ArH), 8.98 (br s, 2H, ArOH), 11.89 (br s, 1H, COOH); ^13^C NMR (300 MHz, DMSO-d_6_): δ 24.4, 28.5, 28.7, 28.8, 29.0, 30.6, 33.6, 35.2, 99.9, 106.2, 144.1, 158.1, 174.4; m/z (ESI): 401.2662 (M^+^ + Na; C_23_H_38_O_4_Na requires 401.2668). **2d**: ^1^H NMR (200 MHz, acetone-d_6_): δ 1.24–1.34 (m, 36H, (CH_2_)_18_), 1.53–1.61 (m, 4H, (CH_2_)_2_), 2.28 (t, 2H, *J* = 7.3 Hz, CH_2_COOH), 2.45 (t, 2H, *J* = 7.5 Hz, CH_2_Ar), 6.18 (s, 3H, ArH); ^13^C NMR (200 MHz, acetone-d_6_): δ 25.7, 28.1, 28.7, 29.1, 29.5, 29.8, 30.04, 30.09, 30.2, 30.4, 30.6, 31.0, 32.1, 34.2, 36.6, 100.9, 107.7, 145.9, 159.3, 174.6; m/z (ESI): 463.3782 (M^+^ + H; C_29_H_51_O_4_ requires 463.3787).

# References

1. Parang, K.; Knaus, E.; Wiebe, L. I.; Sardari, S.; Daneshtalab, M.; Csizmadia, F. *Arch. Pharm. Pharm. Med. Chem.* **1996**, *329*, 475–482. doi:[10.1002/ardp.19963291102](http://dx.doi.org/10.1002/ardp.19963291102)
2. Garigibati, R. S.; Freyer, A. J.; Whittle, R. R.; Weinreb, S. M. *J. Am. Chem. Soc.* **1984,** *106,* 7861–7867. doi:[10.1021/ja00337a036](http://dx.doi.org/10.1021/ja00337a036)
3. Brown, G. D. *J. Nat. Prod.* **1992,** *55,* 1756–1760. doi:[10.1021/np50090a006](http://dx.doi.org/10.1021/np50090a006)
4. Arisawa, M.; Ohimura, K.; Kobayashi, A.; Morita, N. *Chem. Pharm. Bull.* **1989,** *37,* 2431–2434.
5. Kamal-Eldin, A.; Pouru, A.; Eliasson, C.; Åman, P. *J. Sci. Food Agric.* **2000,** *81,* 353–356. doi:[10.1002/1097-0010(200102)81:3<353::AID-JSFA826>3.0.CO;2-X](http://dx.doi.org/10.1002/1097-0010(200102)81:3<353::AID-JSFA826>3.0.CO;2-X)
6. Suzuki, Y.; Esumi, S.; Uramoto, M.; Kono, Y.; Sakurai, A. *Biosci, Biotechnol, Biochem.* **1997,** *61,* 480–486.
